# Effects of a novel externally rotated trochlear design on patellofemoral kinematics in total knee arthroplasty: A cadaveric study

**DOI:** 10.1002/jeo2.70441

**Published:** 2025-10-13

**Authors:** Tommaso Bonanzinga, Alberto Favaro, Luca Bertolino, Gabriele Fassina, Bharat Sharma, Francesco Iacono

**Affiliations:** ^1^ IRCCS Humanitas Research Hospital Rozzano Milan Italy; ^2^ Department of Biomedical Sciences Humanitas University Pieve Emanuele Milan Italy; ^3^ Humanitas University Pieve Emanuele Milan Italy; ^4^ Department of Electronics, Information and Bioengineering Politecnico di Milano Milano Italy; ^5^ Dakshas Foundation Hyderabad India

**Keywords:** knee, patellofemoral joint, patellofemoral kinematics, patellofemoral tracking, total knee arthroplasty

## Abstract

**Purpose:**

To investigate whether a novel femoral component with a trochlear sulcus externally rotated (EXT) by 3°, mimicking the natural relationship between the posterior condylar axis (PCA) and epicondylar axis (EA), improves patellofemoral (PF) kinematics in total knee arthroplasty (TKA) by more closely replicating native patellar motion.

**Methods:**

A cadaveric study was conducted on 12 lower limbs from six fresh‐frozen hemi‐body specimens. After acquiring native knee kinematics using an optical navigation system, each knee underwent TKA with two prosthetic designs: a standard (STD) implant and an EXT variant with a 3° lateralized trochlear groove. Patellar kinematics were evaluated in flexion–extension movements, and quantified via root mean square error, correlation coefficient and normalized range relative to native conditions. Statistical comparisons were performed using the Wilcoxon signed‐rank test.

**Results:**

Patellar flexion was similar across native and prosthetic conditions. However, the STD design showed significantly lower patellar misalignment than the EXT in terms of patellar shift (*p* < 0.01) and exhibited lower inter‐specimen variability. The EXT design resulted in an inversion of patellar tilt patterns and demonstrated reduced correlation with native tilt kinematics. In terms of internal patellar rotation, patterns post‐implantation were comparable between the STD and EXT designs, emerging beyond 50° of flexion. The expected benefits of trochlear external rotation were not confirmed.

**Conclusions:**

Although theoretically promising, the EXT trochlear design did not lead to improved replication of native PF kinematics. The findings suggest that trochlear sulcus lateralization alone is insufficient to restore physiological patellar tracking.

**Level of Evidence:**

Level V, cadaveric study.

**Abbreviations**
CC: correlation coefficient
EA: epicondylar axis
HKA: hip–knee–ankle
KA: kinematic alignment
MA: mechanical alignment
NR: normalized range
PCA: posterior condylar axis
PF: patellofemoral
RMSE: root mean square error
TF: tibiofemoral
TKA: total knee arthroplasty

## INTRODUCTION

In total knee arthroplasty (TKA), orthopaedic surgeons have traditionally employed mechanical alignment (MA) to position the femorotibial (TF) components, aiming to achieve a neutral 180° hip–knee–ankle (HKA) angle by cutting the distal femur and the proximal tibia perpendicular to their respective mechanical axes [9, 26, 27, 30, 47].

In MA, the femoral component is aligned to the axial plane parallel to the epicondylar axis (EA), which is considered the functional axis around which knee flexion and extension occur [4]. Its optimal axial external rotation is crucial to achieve balanced flexion gaps, proper tibiofemoral (TF) and patellofemoral (PF) congruency, thus improving PF tracking [48, 52] and reducing the risk of failure and patellar maltracking.

Nonetheless, intraoperative identification of EA remains a challenge even for experienced surgeons [36]. Conversely, identification of the posterior condylar axis (PCA) during surgery is much easier, allowing the femoral component to be aligned with the EA by applying a fixed 3° of external rotation relative to the PCA.

MA, however, does not reflect patient‐specific anatomical differences, given that a perfectly neutral HKA angle of 180° is rarely observed in the natural alignment of most individuals [17, 26, 27, 40]. Providing such an unnatural correction requires an adaptation from the soft tissue, with a risk of instability and paradoxical kinematics related to variated joint line [12]. These biomechanical alterations may help explain the relatively high rates of knee instability and patient dissatisfaction historically observed when MA was the only available alignment strategy—approximately 20% of patients reported persistent pain or impaired function following TKA.

Nevertheless, kinematic alignment (KA) has emerged as an alternative to MA, aiming to restore the patient's pre‐arthritic joint lines and kinematics, leading to higher patient satisfaction and improved knee function [5, 15, 28, 50, 51]. Our research group has recently developed a method that uses the bisector of the femoral trochlear groove as a novel anatomical landmark for kinematically aligning the femoral component in TKA [29, 41]. This approach aims to streamline the KA procedure, reduce potential sources of error, and achieve outcomes equivalent to KA calliper resections, as described by Howell et al. [28]. One relevant point in KA is that it ensures correct ligament tensioning, eliminating the need for the external rotation of the femoral component [28, 29]. However, without the need for external rotation of the femoral component—originally meant to achieve ligament tensioning in MA—we may lose the benefits of an externally rotated (EXT) trochlear sulcus in terms of improved PF tracking.

In this in vitro study, we investigate whether a newly designed femoral component featuring an EXT trochlear sulcus of 3° (to mimic the relationship between the EA and the PCA) can improve PF kinematics, thereby more closely resembling native PF motion [5].

## MATERIALS AND METHODS

### Set‐up

In this study, the experimental set‐up described and validated by Favaro et al. [20] was used. This set‐up included six fresh‐frozen hemi‐body cadaveric specimens (one male, five females; mean age 87 ± 14.4 years, range 63–101 years; body mass index 17.8 ± 4.9). Each specimen included the pelvis and lower limbs, providing a total of 12 leg samples (l=1,2,…,12). All knee joints were verified to be free from anatomical defects, with intact joint capsules, cruciate and collateral ligaments, and patellar and quadriceps tendons and without a history of prosthetic implant procedures.

For kinematics acquisition, we employed a commercial navigation system (BLU‐IGS, software version 1.4, Orthokey Italia S.r.l.) featuring a bespoke reference frame, specifically designed for intraoperative PF tracking. This frame was attached directly to the patella bone via a medial parapatellar approach, following patella exposure.

### Patellar kinematics

**Figure 1:**
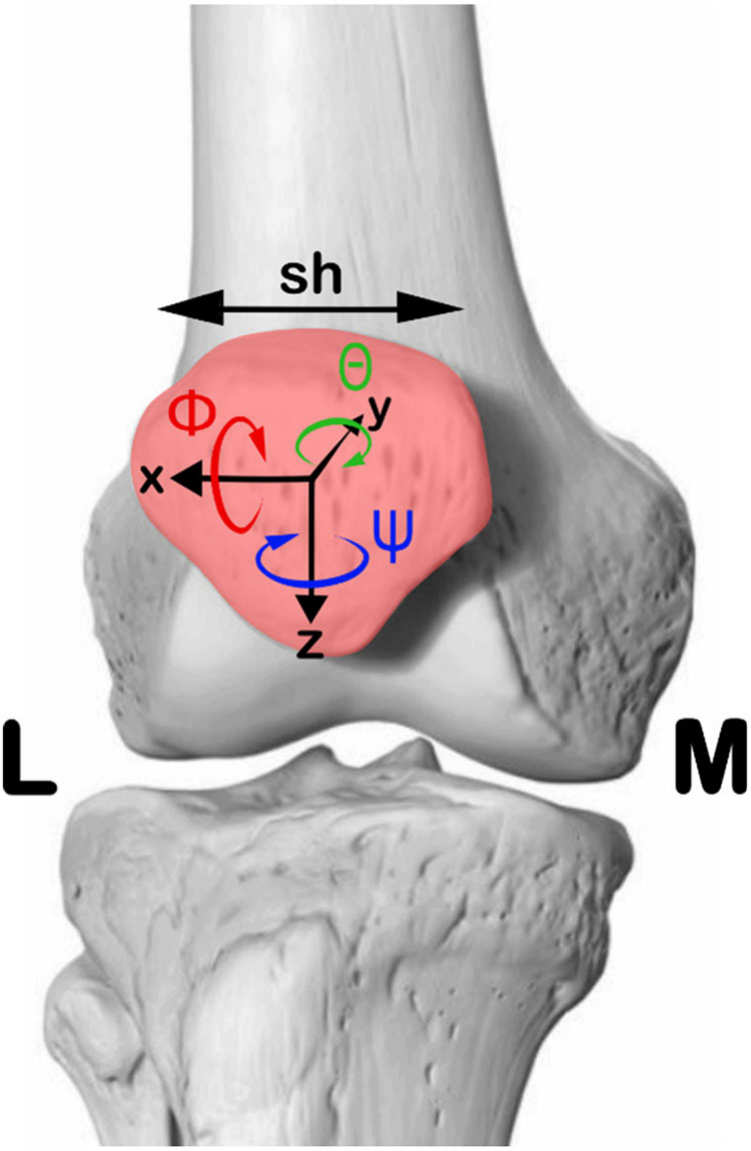
Patella kinematics in terms of rotations and translations. Patella flex‐extension (Φ) is represented in red as a rotation about the *x*‐axis. Patella medial/lateral rotation (θ), in green, happens about the *y*‐axis. Patellar medio‐lateral tilt (ψ) in blue, happens about the *z*‐axis. Medio‐lateral shift (sh) is reported in black. Medial (M) and lateral (L) sides are indicated.

Patellar kinematic parameters were defined using the Grood and Suntay method [24], a widely used approach for the TF joint, adapted to the PF joint. The following patellar motion parameters, as described in Favaro et al. [20] and illustrated in Figure 1, were analyzed:

− Patellar flexion/extension (ϕ): refers to the anterior(+)‐posterior (−) movement of the patella relative to the femur, representing its rotation in the sagittal plane;
− Patellar medial/lateral rotation (θ): occurs when the distal apex of the patella moves laterally (+) or medially (−), indicating rotation in the frontal plane;
− Patellar medial/lateral tilt (ψ): This describes the rotation of the patella's central portion towards the lateral (+) or medial (−) side of the knee, corresponding to movement in the transverse plane; and
− Medial/lateral shift (sh): This represents the translation of the patella towards the lateral (−) or medial (+) side of the knee.

### Experimental campaign

For each leg, the anatomy was acquired through an initial registration phase. Then, the kinematic profiles were recorded separately during knee flexion and extension (α), ranging from $\alpha =0^{\circ }$ (full leg extension) to $\alpha =120^{\circ }$ (full leg flexion).

First, native kinematics were recorded. Then, the TKA procedure was performed, followed by sequential kinematic acquisitions after implanting the two different prosthesis types, featuring the designs reported below.

### Prosthesis designs

Both types of prostheses were 3D‐printed by Rejoint S.r.l., after establishing the most accurate implant size through the software analysis of a preoperative CT scan of the cadaveric knee. This advanced sizing approach, which allows for an uncountable number of implant dimensions, overcomes the traditional concept of a limited set of predefined sizes [10]. The first prosthesis type, which is referred herein as ‘standard’ (STD), follows a conventional trochlear design. The second, referred to as ‘extrarotated’ (EXT), features a trochlear sulcus EXT by 3°, purposely designed to better mimic native PF kinematics.

**Figure 2:**
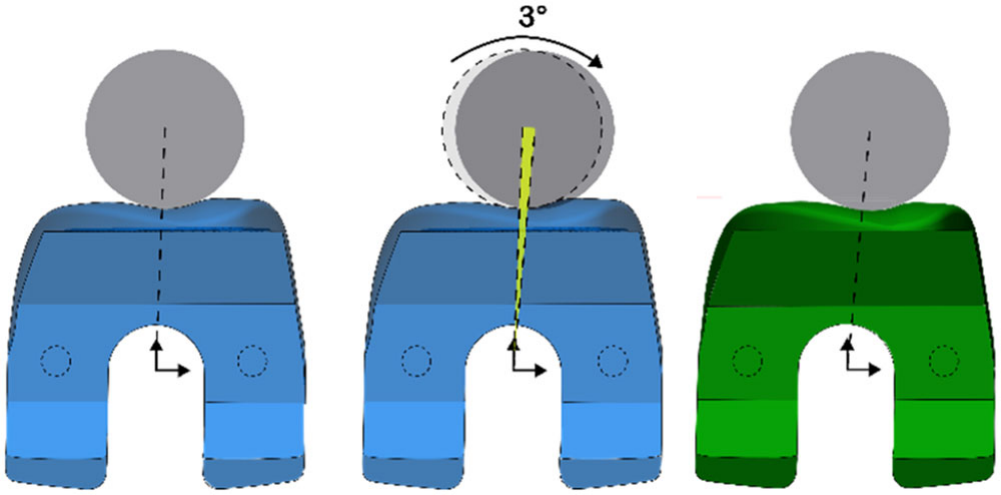
Design of the externally rotated (EXT) version of the prosthesis. The figure to the left shows the standard design with the trochlear sulcus defined by the circle (in grey). The two orthogonal arrows represent the origin of the reference system of the prosthesis (i.e., the origin of the axes). The figure in the middle shows the reference circle rotated by 3° laterally with respect to its origin in order for the trochlear variant to yield 3° of extrarotation of the trochlear sulcus. The figure to the right shows the new design of the lateralized trochlea (i.e., EXT).

In Figure 2, the STD (in light blue) is shown from a superior axial view, having the trochlear sulcus encompassed by a grey circle.

**Figure 3:**
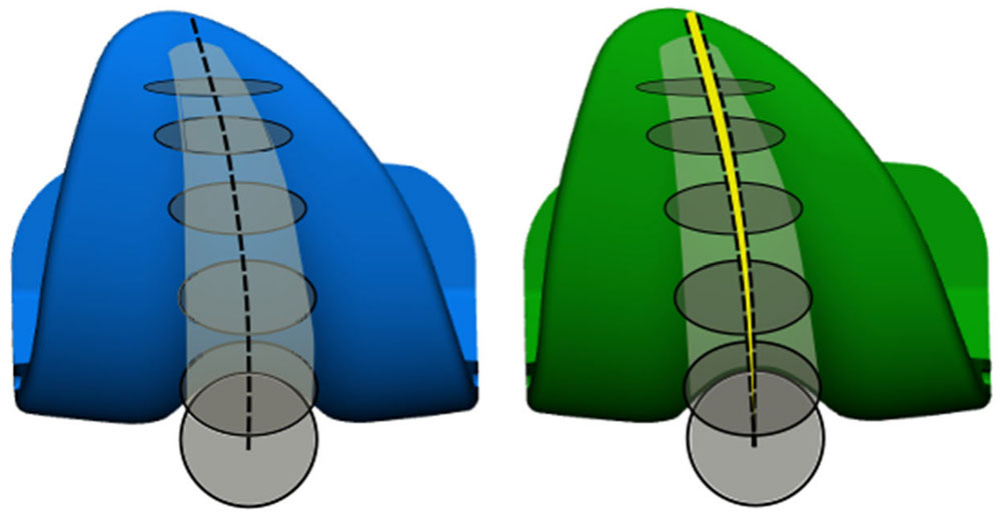
In the coronal view, there is a difference in terms of the trochlear sulcus between the standard design and the externally rotated design.

In the frontal aspect (Figure 3), this circle converts into a cylindrical shape, extending throughout the frontal shield of the prosthesis, from the apical top, down to the intercondylar notch. The external rotation of the trochlear sulcus featured by the EXT design is achieved by rotating this circle around the centre of the trochlea, marked in Figure 2 by the origin of the two black arrows. In the coronal aspect of the femoral component, this rotation results in a lateral displacement of the trochlear sulcus, which increases progressively moving toward the apical part of the prosthesis (see Figure 3).

For each prosthesis, namely STD and EXT, kinematics was acquired on three flexion–extension repetitions. To minimize the risk of bone rupture, the patella was not resurfaced during the procedure. During all measurements, the knee joint capsule was sutured to maintain joint stability throughout the experiment.

### Data analysis

For each cadaveric sample and for all four parameters, the mean trajectory was computed over the three repetitions and considered as reference for the following analyses. By averaging the trajectory of all leg samples, a mean trajectory for each kinematic parameter is computed.

To quantify the dispersion of kinematic measurements across different samples, we introduced the concept of normalized range (NR). As an example, for the parameter of patellar flexion ϕ, NR at different angles of leg flexion (α) is defined (denoted as $\hat{\phi }$) as follows:

$$\hat{\phi }\left(\alpha \right)=\frac{\max\left(\phi_{l}\left(\alpha \right)\right)-\min\left(\phi_{l}\left(\alpha \right)\right)}{\max\left(\left|\bar{\phi_{l}}\right|\right)}\forall \;l\in \{1,2,\ldots ,12\}$$

Specifically, the excursion of ϕ defined as the difference between its maximum value at flexion angle ϕ at flexion angle α across all legs ($\max\left(\phi_{l}\left(\alpha \right)\right))$ and its minimum value ($\min\left(\phi_{l}\left(\alpha \right)\right)$) is normalized by the maximum absolute value of the average signal ($\max\left(\left|\bar{\phi_{l}}\right|\right)$) calculated across the 12 legs.

Similarly, NR was computed for the other kinematics parameters: $\hat{\theta }\left(\alpha \right),\hat{\psi }\left(\alpha \right),\hat{\text{sh}}\left(\alpha \right)$.

Lower NR values indicate that the traces from different subjects are closely aligned, while higher values suggest greater variability across the samples.

To assess the deviation between native (NTV) and prosthetic kinematics (STD and EXT), the following measures have been computed:

− Root mean square error (RMSE): Lower RMSE values indicate greater similarity between the native and prosthetic kinematic profiles, while also quantifying potential offsets.
− Correlation coefficient (CC): Measures the strength and direction of the linear relationship between native and prosthetic kinematics. A high CC value indicates that an increase in a specific kinematic parameter in the native configuration corresponds to an increase in the prosthetic configuration, and vice versa.

RMSE and CC were evaluated across the entire range of motion, that is, from full extension $(\alpha =0^{\circ }),$ to full flexion ($\alpha =120^{\circ }$).

A statistical analysis was conducted to assess the significance of differences in terms of RMSE and CC across the various prosthetic conditions. The Shapiro–Wilk test was applied to evaluate the normality of the computed parameters. As they did not follow a normal distribution, the nonparametric Wilcoxon signed‐rank test was applied to evaluate the significance of the observed differences between the STD and ADV.

## RESULTS

### Kinematic trajectories

**Figure 4:**
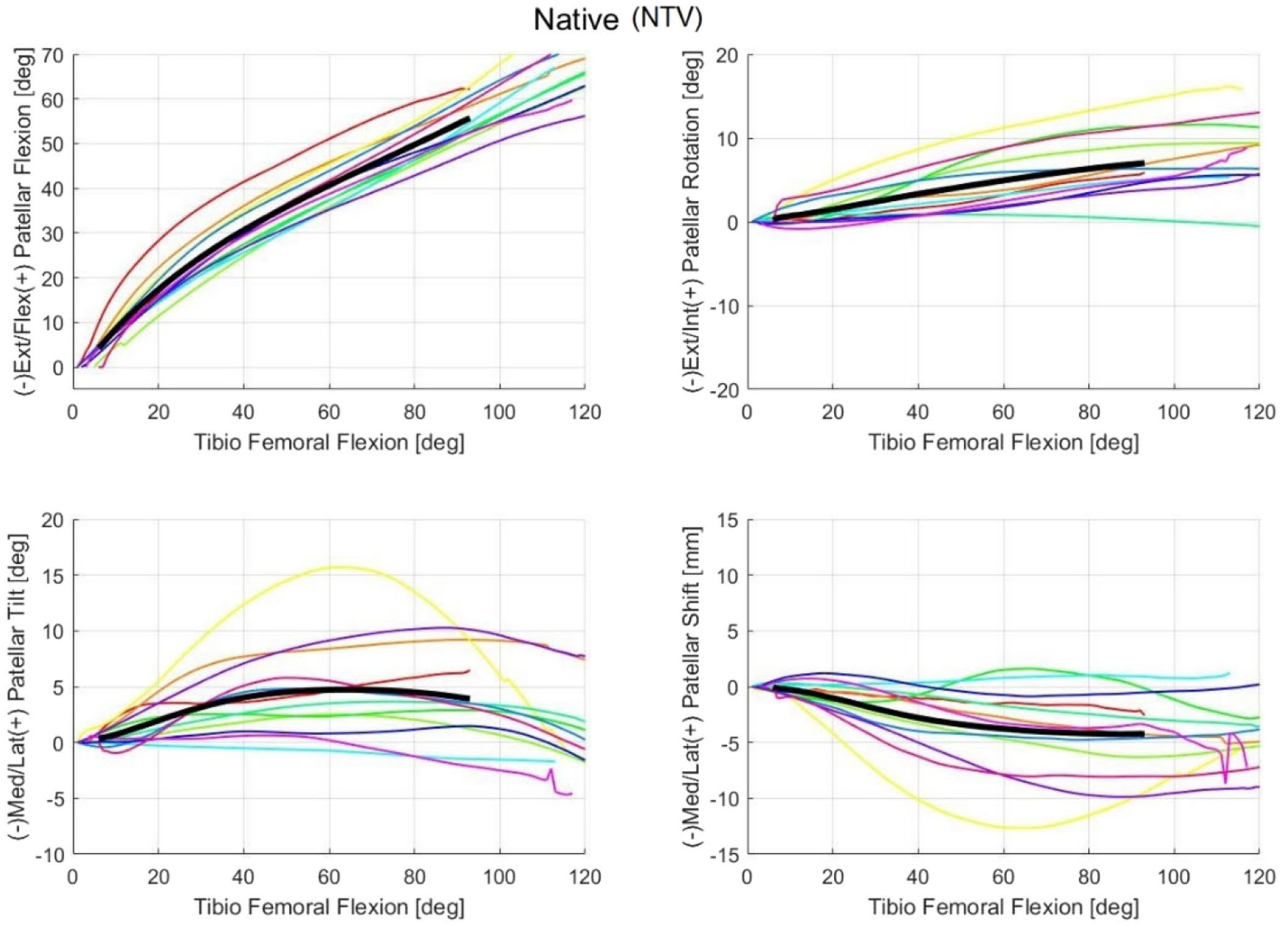
The coloured lines represent the mean trajectories for each cadaveric sample, reported for each patellar kinematic parameter of the native (NTV) condition. Additionally, the black line traces the averaged trajectory measured across the different samples for each kinematic parameter.

**Figure 5:**
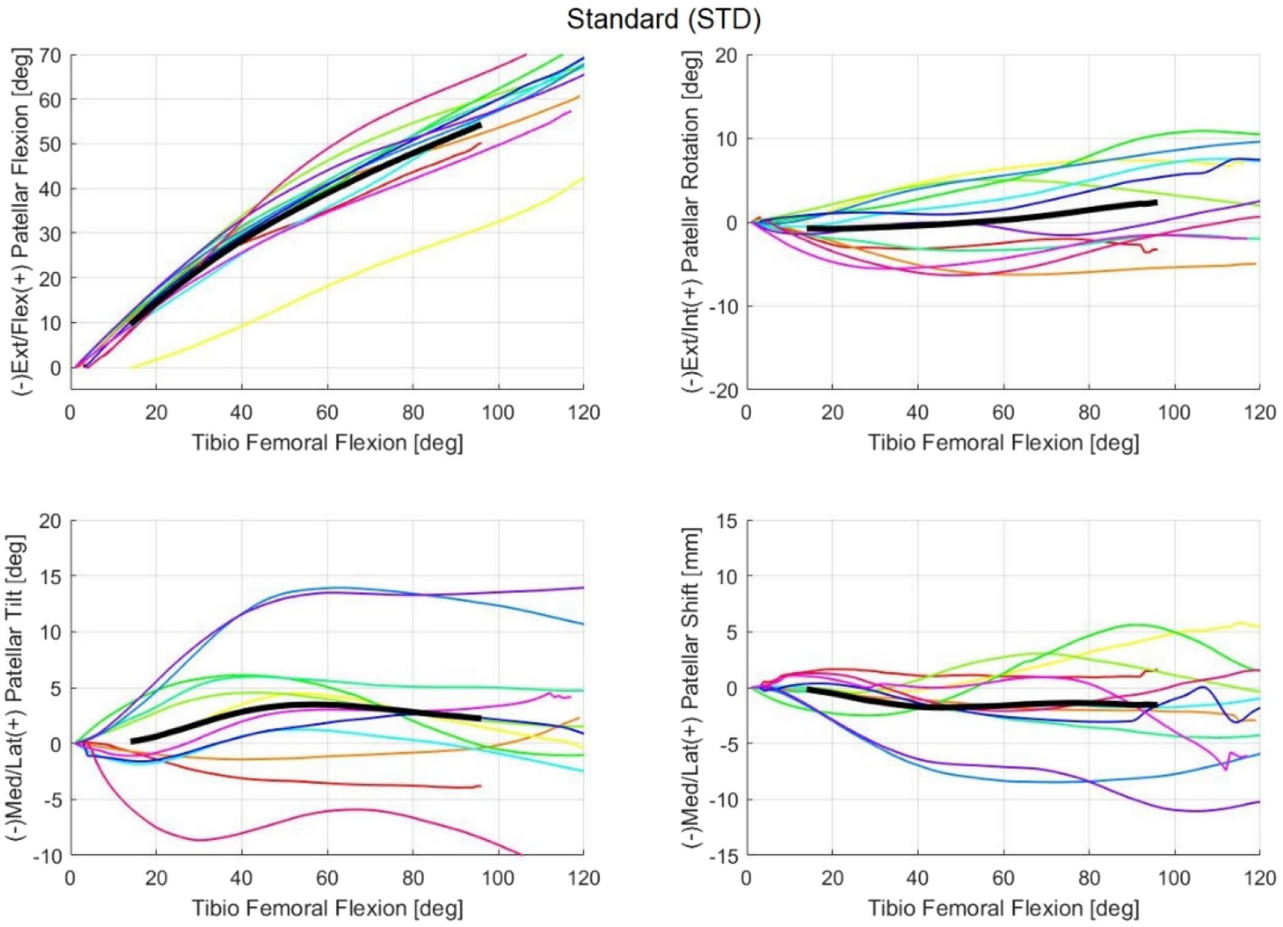
The coloured lines represent the mean trajectories for each cadaveric sample and were traced for each patellar kinematic parameter of the standard (STD) condition. The black line traces the averaged trajectory measured across the different samples for each kinematic parameter.

**Figure 6:**
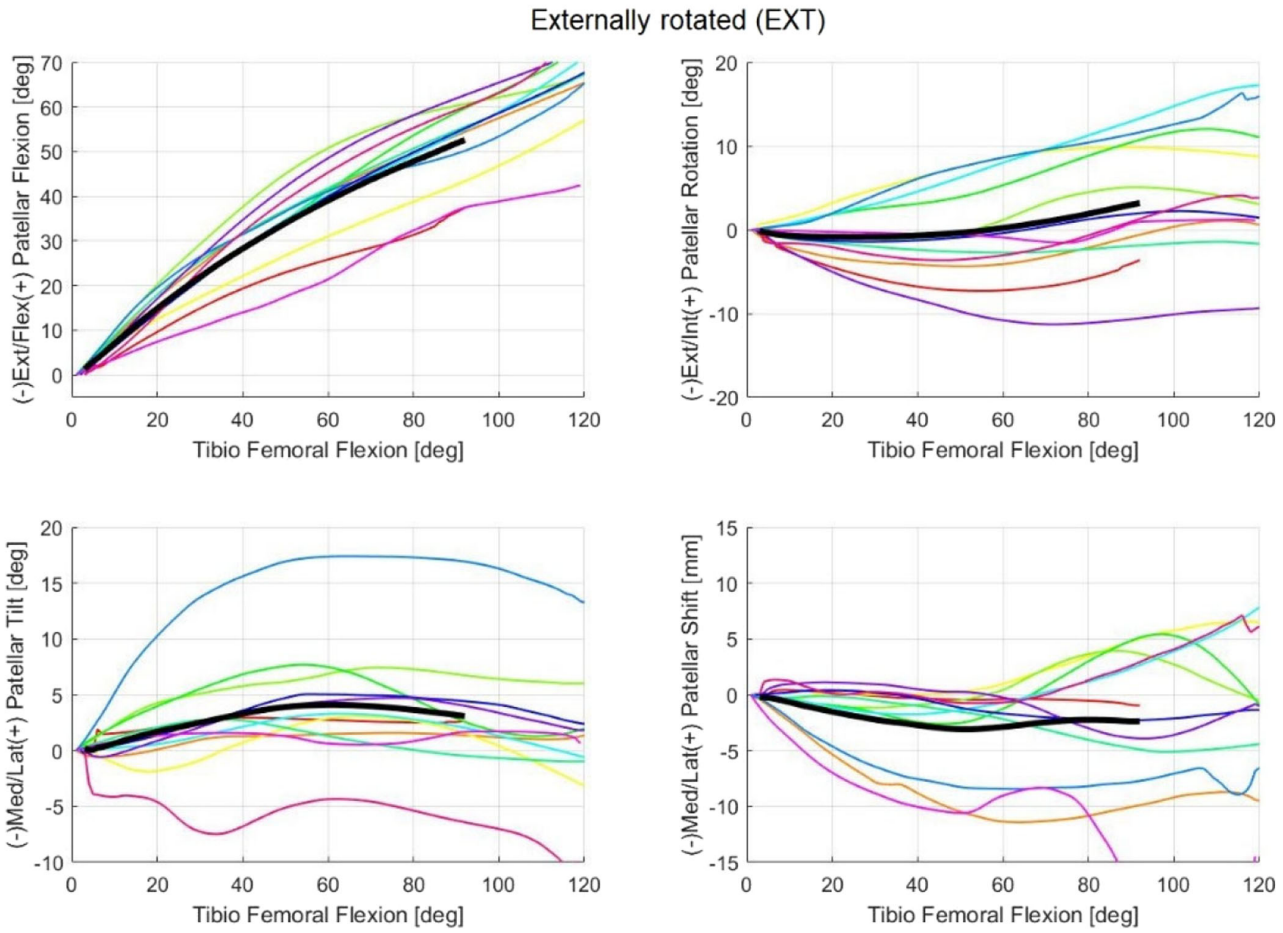
The coloured lines represent the mean trajectories for each cadaveric sample, reported for each patellar kinematic parameter of the externally rotated (EXT) condition. The black line traces the averaged trajectory measured across the different samples for each kinematic parameter.

For each cadaveric sample, the mean trajectories calculated over the three flexion–extension repetitions were reported for each patellar kinematic parameter in the NTV, STD and EXT conditions (Figures 4, 5, 6, respectively). The full range of motion (*α* = 0° to *α* = 120°) is covered. However, for some subjects, data beyond 90° of flexion were unavailable; consequently, the overall trajectory (i.e., across all samples) was computed only up to *α* = 90° to ensure consistency across samples (reported in black in Figures 4, 5, 6).

Additionally, the data acquired for Leg 3 (represented by the yellow line in the figures) consistently fall outside the confidence interval (defined as two standard deviations) for most measurements, even in native conditions. For this reason, Leg 3 was considered an outlier and has been excluded from further analysis. A plausible explanation for this abnormal behaviour could be a technical issue during the fixation of the patellar reference frame and its subsequent mobilization, potentially compounded by an improper closure of the joint capsule. These factors may have prevented the patella from correctly engaging the trochlear groove throughout the range of motion, resulting in a looser tracking pattern more susceptible to the influence of soft tissues and gravity. This interpretation is supported by the observation that the contralateral limb from the same specimen did not display similar deviations, suggesting a localized procedural issue rather than an anatomical anomaly.

### Intra‐sample variability

Since three acquisitions were performed for each leg, intra‐sample variability was assessed by calculating the mean standard deviation for each kinematic parameter and in each experimental condition (NTV, STD and EXT). Median and interquartile range of the mean standard deviation across the 12 legs are reported in Table 1.

**Table 1:** Intra‐sample variability.

	Flexion (°)	Rotation (°)	Tilt (°)	Shift (mm)
NTV	0.43 (0.29)	0.33 (0.34)	0.20 (0.29)	0.35 (0.30)
STD	0.53 (0.25)	0.39 (0.27)	0.41 (0.17)	0.67 (0.55)
EXT	0.63 (0.60)	0.38 (0.26)	0.45 (0.50)	0.54 (0.28)

Abbreviations: EXT, externally rotated; NTV, native; STD, standard.

### Inter‐sample variability

The NR was applied to ensure consistency of the identified parameters across the 12 legs available. Table 2 reports mean NR values obtained for each kinematic parameter in each condition, for both leg flexion and leg extension. It should be noted that the measurements obtained in the extension phase show a lower NR, that is, a higher repeatability, than during flexion, although the difference is not statistically significant at the Wilcoxon test (*p* = 0.13). Data analysis was also performed for the flexion, yielding similar conclusions, though with reduced statistical significance due to higher intragroup variability. Here, we report results for the knee extension phase alone.

**Table 2:** Normalized range index for each kinematic parameter in each prosthesis.

Condition	Measurement	Flexion	Extension
NTV	Patellar flexion	1.11	1.05
	Patellar rotation	1.61	1.62
	Patellar tilt	2.02	2.06
	Patellar shift	2.21	2.05
STD	Patellar flexion	1.14	1.05
	Patellar rotation	9.14	5.21
	Patellar tilt	2.96	3.84
	Patellar shift	3.37	5.24
EXT	Patellar flexion	1.18	1.16
	Patellar rotation	11.05	5.19
	Patellar tilt	4.85	2.84
	Patellar shift	4.51	4.54

*Note*: The first column represents the values obtained during knee flexion, while the second column refers to knee extension.

Abbreviations: EXT, externally rotated; NTV, native; STD, standard.

### Prostheses comparison

**Figure 7:**
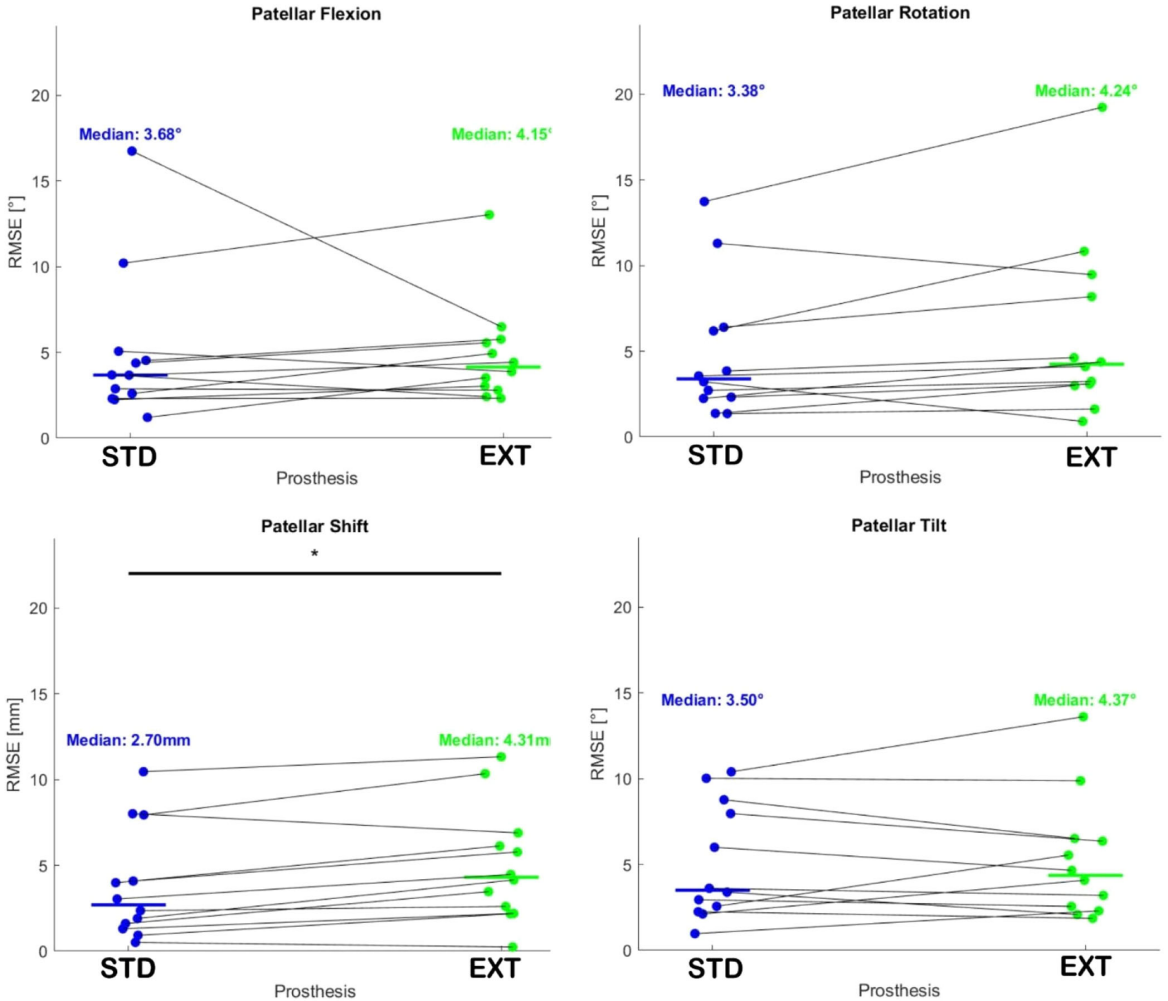
Scatterplot of the root mean square error (RMSE) computed for the two prostheses, STD and EXT, with respect to the native kinematics. The solid line represents the median value. * indicates the statistical significance computed at the Wilcoxon test. EXT, externally rotated; STD, standard.

The RMSE and CC were used for the comparison. Figure 7 illustrates the RMSE computed for the two prostheses with respect to the native kinematics for all kinematic parameters. Notably, the STD demonstrates superior performance, with lower median RMSE values across all four kinematic parameters compared to the EXT. Specifically, this difference is statistically significant at the Wilcoxon test for the parameter of patellar shift (*p* value < 0.01).

**Figure 8:**
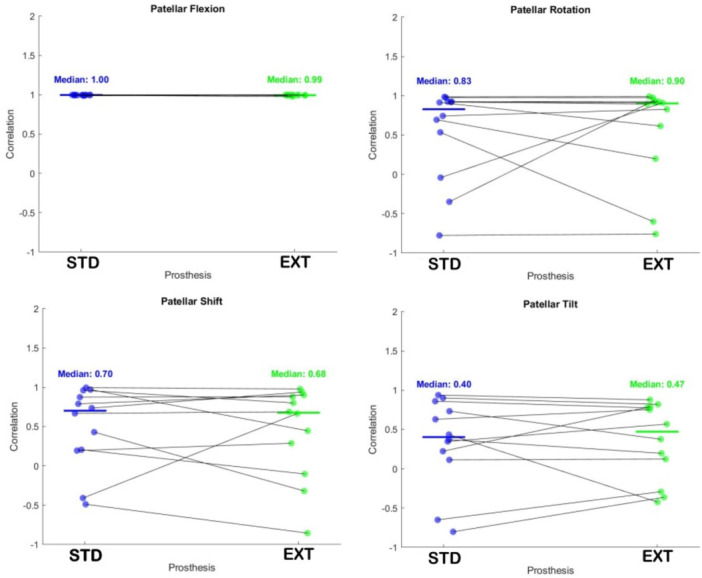
Scatterplot of the correlation coefficient computed for two prostheses, STD and EXT, with respect to the native kinematics. The solid line represents the median value. EXT, externally rotated; STD, standard.

Figure 8 reports the CC of each kinematic parameter compared to the native kinematics. Median values appear comparable for the two prostheses, STD and EXT, in all the conditions, although the STD generally exhibits smaller variance than the EXT. Moreover, except for the outliers, the correlation values for the STD are positive for all kinematic parameters, unlike the EXT for which the patellar tilt shows negative correlation, suggesting that for this parameter EXT's kinematic is inversed with respect to the NTV. No statistically significant difference was found among the different samples in the Wilcoxon test.

## DISCUSSION

The objective of this cadaveric study was to evaluate a newly designed femoral component featuring a trochlear sulcus EXT by 3°, in order to determine whether it more accurately replicates native PF motion and thereby improves PF kinematics. Unlike previous studies [2, 25, 40, 41, 43, 44, 46], where implant design directly couples the trochlear groove position to the TF flexion gap, this study investigates the effects of decoupling the PF component from the flexion gap mechanism.

Since an external rotation of 3° is believed to improve patellotrochlear articulation, the current implant incorporates a trochlear groove EXT in the axial plane, effectively achieving lateralization of the trochlear sulcus. This modification allows for a reduced lateral sulcus angle while maintaining patellar engagement—aligning with previous findings that demonstrated lateralization of the native trochlear groove in the coronal projection [11, 40].

Although several previous in vivo and in vitro studies have investigated PF kinematics following TKA [1, 3, 7, 13, 14, 31, 35, 37, 39, 41, 43, 44, 45], most report persistent discrepancies in extension kinematics compared to the native knee—findings corroborated by our current data. Results on the averaged patellar flexion were comparable between the native knee and the two prosthetic designs. However, when looking at the other kinematics parameters,the STD implant exhibits significantly less patellar misalignment with respect to the native kinematics compared to the EXT design, particularly in terms of patellar shift, where statistical significance was achieved. Furthermore, the STD implant showed reduced variability among specimens, except in patellar tilt, and a stronger correlation with native anatomy. In contrast, the EXT configuration appeared to reverse patellar tilt patterns.

As to patellar shift, notable contrast emerged between the findings of Keshmiri et al. [32] and the current study, although both used similar experimental set‐ups and navigation systems. In our native knee samples, a continuous medial shift was observed from 30° to 90° of flexion, whereas they reported medialization throughout flexion, followed by terminal lateralization between 80° and 90°. Post‐TKA, our data showed medial shift through flexion with an inflection point beyond 40° in the STD group. In contrast, Keshmiri et al. [32] recorded a 2.5–3 mm medial shift in the native patella post‐TKA, which increased to 3–5 mm following resurfacing. The magnitude of shift in our study aligns with data from Tanikawa et al. [52], who also observed increasing shift with flexion and reduced shift magnitude after prosthesis implantation. Moreover, the waveform of patellar shift for the STD implant closely matched the patterns described by Barnik et al. [6].

The patellar tilt magnitudes observed in this study were consistent with those reported by Tanikawa et al. [52], where the native knee demonstrated greater tilt than prosthetic knees. Similarly, the waveform of patellar tilt in both native and prosthetic knees mirrored findings by Barnik et al. [6]. Specifically, lateral patellar tilt decreased from 60° in the native knee and from 50° in both EXT and STD implants. This may suggest a possible influence of medial PF ligament laxity during terminal extension [22] or patellar component medialization [42]. Keshmiri et al. [32] described a continuous increase in lateral tilt from 30° to 90° of flexion in the native knee and from 50° to 90° in the TKA knee with a native patella, whereas lateral tilt decreased from 50° to 90° after resurfacing.

In native knees, internal patellar rotation tends to increase progressively with flexion. In contrast, all prostheses showed neither internal nor external rotation up to 50° of flexion. After this point, internal rotation appears. This is in line with Keshmiri et al. [32] who also reported an increase in internal rotation from 50° to 90°.

Several other factors could have influenced the differences observed between the current and prior studies. These include changes in patellar tendon fibre orientation [8], condyle‐trochlear angle [37], type of trochlea [16, 53], distal trochlear sulcus angle [11, 21] mismatch between native and prosthesis trochlea [49], posterior condylar angle [1, 19], trochlear groove orientation to PCA [1, 23], cage for trochlear implant [40] and rotation profile of the limb [33]. Each of these may act via the patellar tendon or posterior cruciate ligament in the native knee.

Customizing implants to match individual trochlear morphology remains a significant challenge [49].

Multiple studies have shown that gap balancing techniques—used to define posterior femoral resection—improve restoration of PF kinematics following TKA [18, 26, 34, 37, 38]. This cadaveric study investigated whether a femoral component with a trochlear sulcus EXT by 3° could improve PF kinematics in TKA by more closely replicating native patellar motion. The findings demonstrate that, while patellar flexion was comparable across native and prosthetic conditions, the STD design outperformed the EXT variant, particularly in terms of accuracy in replicating the patellar shift and inter‐specimen variability. Notably, the EXT configuration showed an inversion of patellar tilt behaviour. Therefore, while the concept of decoupling trochlear orientation from the TF flexion gap remains of interest and despite a theoretical rationale supporting the lateralization of the trochlear groove, our data indicate that an EXT trochlea alone is not sufficient to restore physiological PF kinematics. Further refinements may be necessary to optimize PF function following TKA.

### Study limitations

These results must be interpreted considering several limitations, in addition to the relatively small sample size. The most significant constraint is the unavailability of kinematic data for knee flexion angles beyond 90° in all cases, as well as the lack of dynamic loading conditions during data acquisition. Moreover, it is not possible to draw definitive conclusions or formulate robust hypotheses regarding the potential differences in patellar kinematics in the presence of patellar resurfacing. Other studies in the literature considered patella resurfacing [37, 46]; however, they were not in a format directly comparable with our results due to non‐standardized study design. The inclusion of the patella resurfacing as an additional surgical variable would introduce a substantial confounding factor, requiring a different and dedicated experimental set‐up, as well as a tailored methodological approach. However, despite the patella resurfacing condition being beyond the scope of the present study, its exclusion nonetheless represents a limitation.

## CONCLUSIONS

This cadaveric study evaluated whether a novel femoral component with a trochlear sulcus EXT by 3°, mimicking the anatomical relationship between the epicondylar and posterior condylar axes, could better replicate native PF kinematics in TKA. Despite its theoretical rationale, the STD design showed superior performance in terms of patellar shift accuracy, lower inter‐specimen variability, and closer alignment with native kinematics.

These results suggest that isolated external rotation of the trochlear groove, though biomechanically intuitive, is insufficient to address the complex determinants of PF motion and does not restore physiological kinematics after TKA. Further research is needed to explore more comprehensive design strategies that integrate component geometry with individualized alignment protocols and soft tissue considerations to optimize PF function postoperatively.

## AUTHOR CONTRIBUTIONS

*Conceptualization*: Tommaso Bonanzinga, Alberto Favaro and Francesco Iacono. *Validation*: Alberto Favaro and Francesco Iacono. *Formal analysis*: Gabriele Fassina. *Investigation*: Tommaso Bonanzinga, Alberto Favaro, Luca Bertolino and Francesco Iacono. *Data curation*: Alberto Favaro and Gabriele Fassina. *Writing—original draft preparation*: Alberto Favaro, Luca Bertolino, Gabriele Fassina and Bharat Sharma. *Writing—review and editing*: Alberto Favaro and Luca Bertolino. *Project administration*: Francesco Iacono. *Supervision*: Luca Bertolino and Francesco Iacono. *Funding acquisition*: Luca Bertolino and Francesco Iacono. All authors have read and agreed to the published version of the manuscript.

## CONFLICT OF INTEREST STATEMENT

The authors declare no conflicts of interest.

## ETHICS STATEMENT

The study was approved on 17 March 2022 by the Institutional Review Board of Humanitas Research Hospital with approval code: 354/22.

## Data Availability

The data sets presented in this article are not readily available because the data are part of an ongoing study. Requests to access the data sets should be directed to the corresponding author.
